# Deciphering the QR Code of the CRISPR-Cas9 System: Synergy between Gln768 (Q) and Arg976 (R)

**DOI:** 10.1021/acsphyschemau.2c00041

**Published:** 2022-09-22

**Authors:** Vangelis Daskalakis

**Affiliations:** Department of Chemical Engineering, Cyprus University of Technology, 95 Eirinis Street, 3603 Limassol, Cyprus

**Keywords:** CRISPR-Cas9, molecular dynamics, Markov state model, machine learning, mutants

## Abstract

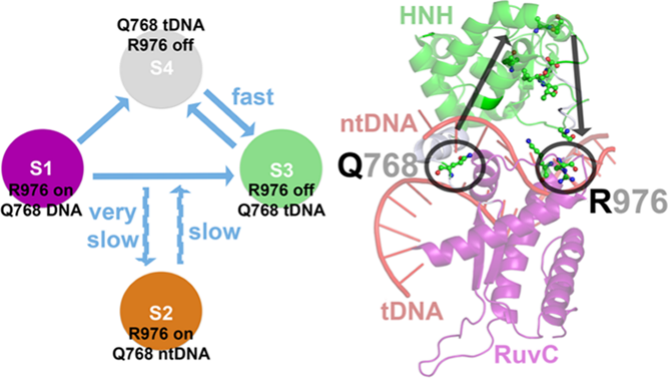

Markov state models (MSMs) and machine learning (ML) algorithms can extrapolate the long-time-scale behavior of large biomolecules from molecular dynamics (MD) trajectories. In this study, an MD–MSM–ML scheme has been applied to probe the large endonuclease (Cas9) in the bacterial adaptive immunity CRISPR-Cas9 system. CRISPR has become a programmable and state-of-the-art powerful genome editing tool that has already revolutionized life sciences. CRISPR-Cas9 is programmed to process specific DNA sequences in the genome. However, human/biomedical applications are compromised by off-target DNA damage. Characterization of Cas9 at the structural and biophysical levels is a prerequisite for the development of efficient and high-fidelity Cas9 variants. The Cas9 wild type and two variants (R63A–R66A–R70A, R69A–R71A–R74A–R78A) are studied herein. The configurational space of Cas9 is provided with a focus on the conformations of the side chains of two residues (Gln768 and Arg976). A model for the synergy between those two residues is proposed. The results are discussed within the context of experimental literature. The results and methodology can be exploited for the study of large biomolecules in general and for the engineering of more efficient and safer Cas9 variants for applications.

## Introduction

The 2020 Nobel Prize in Chemistry was awarded among others for the discovery and application of the bacterial adaptive immunity clustered regularly interspaced short palindromic repeats (CRISPR) system. This widely used exciting technology involves a single protein of 1368 residues called Cas9 (**C**RISPR-**as**sociated) with two endonuclease domains for double-stranded DNA (dsDNA) cleavage. The CRISPR-Cas9 system has become a programmable and state-of-the-art powerful genome editing tool that has already revolutionized the biomedical and pharmaceutical fields and the fundamental research in life sciences.^1,2^ It has been successfully repurposed to become the forefront technology for genome manipulation and live-cell imaging in basic and applied research, with ease of design, minimum requirements, and simplicity of application.^2−5^ The field is rapidly evolving with promising applications also in the inactivation of oncogenes, activation of cancer suppressor genes, and the treatment of viral infections.^2^

The Cas9 from *Streptococcus pyogenes* (SpCas9) has been most studied.^2^ Cas9 first identifies the dsDNA target via a short sequence of 2–5 nucleotides within the DNA called a protospacer adjacent motif (PAM). CRISPR-Cas9 is programmed to process the dsDNA sequences in the genome with complementarity to a 20 nucleotide (nt) spacer sequence of a CRISPR RNA (crRNA) of either the *trans*-activating crRNA (tracrRNA) in complex with crRNA or a tracrRNA–crRNA fused complex called single-guide RNA (sgRNA) of around 100 nt bound to the Cas9 protein scaffold.^6^ Partial complementarity between crRNA and the target DNA strand (tsDNA) is tolerated by the Cas9 recognition mechanism. Thus, the CRISPR-Cas9 safe use as a genome editing tool in clinical or therapeutic applications for currently uncured genetic-based diseases is compromised by off-target DNA cleavage and large deleterious structural chromosomal variants that can be passed on to the next generations and disrupt gene function or regulation.^6−11^ Off-target DNA cleavage refers to unintended mutations in the genome (outcomes) at sites other than the targeted one, and while it provides a considerable advantage for bacteria to fight phage variants or viral escape mutants, it can become detrimental for applications in human genome editing with undesired phenotypes.^8,12^ The drawbacks in the application of the CRISPR-Cas9 tool certainly do not mean that the CRISPR-Cas9 gene editing tool should not be employed. On the contrary, it is critical that research should focus on identifying residues that influence the Cas9 specificity as an important step toward reducing the adverse effects of unintended or undetected mutations in the cells of interest and the target system in general.^13^ The ideally engineered Cas9 should prevent cleavage of DNA in the presence of only one bp mismatch. There are several Cas9 engineered highly specific variants with reduced off-target effects,^13−16^ the majority of which however exhibits severely reduced cleavage rates, even at on-target DNA sites.^13,17,18^

**Figure 1:**
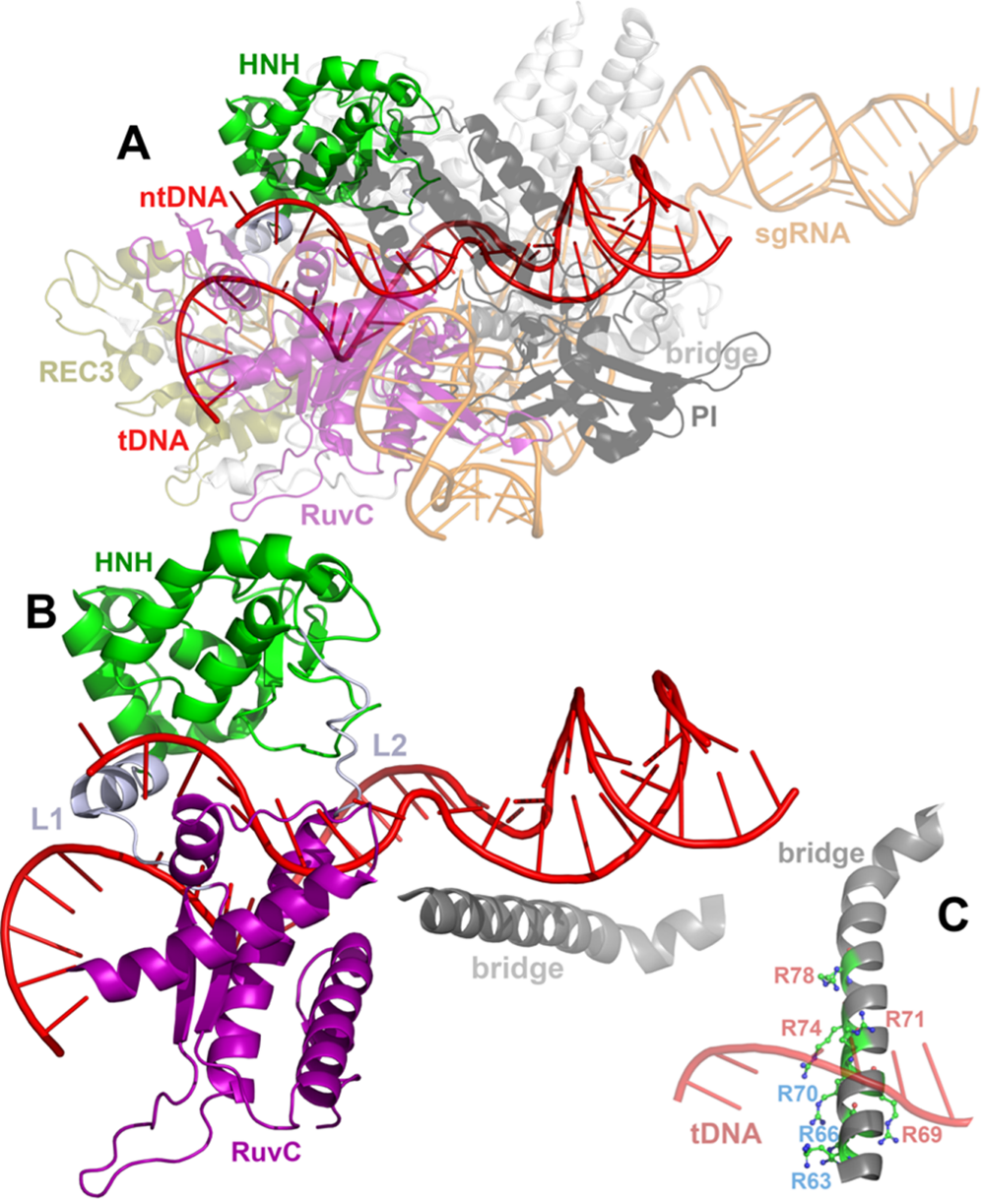
Structure of the Cas9 from *S. pyogenes* (pdb: 5f9r) (A). All Cas9 domains are shown color-coded along with labels of the same color. The double-strand DNA is shown in a red cartoon, and the sgRNA is shown in an orange cartoon. (B) Selected domains of Cas9 are shown in cartoon representation (the 718–1001 region with HNH, part of RuvC, and the linkers L1–L2; the bridge domain). (C) Cas9 bridge domain is shown enlarged with selected Arg residues (*R*) for clarity; blue labels refer to the R63A–R66A–R70A mutated group of residues, and red labels refer to the R69A–R71A–R74A–R78A group of mutated residues.

The most complete crystal structure of the Cas9–sgRNA–dsDNA system (SpCas9) is shown in Figure 1A,^19^ with endonuclease domains HNH and RuvC. HNH is the endonuclease domain of SpCas9 (residues 779–906) for the tsDNA that contains the catalytic His840. RuvC is the second endonuclease domain of SpCas9 (residues 1–59, 718–764, and 917–1098) with the catalytic His983 that cleaves the nontarget strand DNA (ntDNA). Arg976 belongs to the RuvC endonuclease domain that contacts the scissile phosphate and stabilizes the active complex by a “down” conformation toward the active site, with its positive side chain in close contact with the scissile phosphate.^20^ In detail, in the latter configuration, Arg976 is positioned with its side chain toward two catalytic Mg^2+^ atoms 4.2 Å apart.^13,20^ In the inactive conformation, Arg976 interacts with Gln910, Leu911, and Lys913 of the HNH domain.^20^ Residues Arg63, Arg66, and Arg70 of the Cas9 bridge domain (Figure 1B,C) reduce the Cas9 specificity by stabilizing the R-loop structure (sgRNA–DNA hybrid) even in the presence of mismatches in PAM-adjacent sites. Gln768, located at the HNH–RuvC border, is involved in the sensitivity to mismatches in the PAM-distal site and especially a reduced specificity to a mismatch at position 15, whereas Arg69, Arg71, Arg74, and Arg78 bridge residues render the protein more sensitive to mismatches and they are involved in the increase of Cas9 specificity.^21^ The arginine residues in the Cas9 bridge domain (residues 60–93) influence both the binding of nucleic acid helices and are also essential for the denaturation of dsDNA.^21,22^ Moreover, a Q768A–R63A dual mutant has exerted improved specificity of Cas9.^21^ Mutations that affect the signal transmission from the REC domain to RuvC (Figure 1A), like K855A, K810A, and K848A, are important for the Cas9 specificity enhancement.^23^ The REC3 domain is responsible for sensing sgRNA–DNA mismatches.^15,24^ Thus, many Cas9 residues balance between specificity and mismatch tolerance for the natural bacterial CRISPR immune system.^21^ Characterization of Cas9 at the structural and biophysical levels is a prerequisite for the molecular engineering of Cas9 toward the increase of both the specificity and efficiency of this enzyme to prevent the onset of off-target effects. Although the K855A, K810A, and K848A mutations are well characterized both experimentally and computationally,^23^ an atomic-scale insight into the dynamics of the R63A, R66A, R70A, R69A, R71A, R74A, R78A mutations in the bridge domain of Cas9 is lacking. Herein, we seek to find whether these mutations also alter the allosteric communication between the catalytic domains. These mutations determine the sensitivity to mismatches along the sgRNA–DNA hybrid duplex^21^ and are probed herein by computational approaches like classical molecular dynamics (MD), Markov state modeling (MSM),^25,26^ an enhanced sampling technique,^27,28^ and machine (deep) learning algorithms.^29−31^ We focus on the dynamics of the catalytic His840 (HNH) and Arg976 (RuvC) residues, along with the Gln768 dynamics belonging to the linker domain between HNH and RuvC for the Cas9 wild type and mutants. The Arg976/Gln768 dynamics are also determined in relation to the presence of Mg^2+^ ions that are indispensable for the action of Cas proteins.^32^ The long-time-scale behavior described hereafter refers only to a small part of Cas9, either to the 718–1001 Cas9 region (MSM) or to the 767–984 Cas9 region (machine learning analysis).

## Results and Discussion

### Classical Molecular Dynamics and Markov State Model

The MD method has been widely used to study biomolecular interactions, as in the case of Cas9.^20,33−37^ To the best of our knowledge, most of the computational studies in the literature have focused so far on the wild-type (wt) Cas9, or the effect of base pair (bp) mismatches between sgRNA and tDNA. Similar to the study by Nierzwicki et al.,^23^ our working hypothesis is that mutations in the bridge domain of Cas9, e.g., R63A, R66A, R70A, R69A, R71A, R74A, R78A (Figure 1C), along with different concentrations of Mg^2+^ should induce conformational changes in the Cas9 protein scaffold that affect its mechanism of action, cleavage rate, and specificity but more importantly the allosteric communication toward the catalytic domains.^21,32^ Thus, such external stimuli to the protein have been chosen to sample part of the configurational space of Cas9 within the MD method. Three Cas9–sgRNA–dsDNA variants have been prepared; the wt from *S. pyogenes* and its two R63A–R66A–R70A and R69A–R71A–R74A–R78A mutants, with both sgRNA and dsDNA bound for cleavage, based on the most complete resolved crystal structure of a Cas9–sgRNA–dsDNA system.^19^ The systems were hydrated, and different concentrations of Mg^2+^ (low, high) were added. In total, six systems were built, and six classical MD trajectories were run respectively for 1 μs each at 303 K. Please refer to the Materials and Methods section for further details.

To adequately characterize the structural dynamics of Cas9, a combination of the all-atom MD simulations with Markov state model (MSM) theory is applied.^26,38,39^ This enables the extraction of long-time-scale dynamics from rather short-time-scale MD trajectories. The application and accuracy of the powerful MSM theory have been demonstrated in many cases also by experiments that include protein–protein or protein–drug binding kinetics, as well as protein folding rates, protein dynamics, and long-time-scale protein conformations (macrostates).^40−43^ A relatively large protein like Cas9 (∼160 kDa, 1368 residues) has multiple domains that work in synergy for the recognition and cleavage of dsDNA.^2,44^ However, for the MSM models, this study has focused on the backbone atoms of the 718–1001 residue region that contains the HNH domain (catalytic His840), part of the RuvC domain (catalytic Arg976, His983), and the L1/L2 linkers (Figure 1B). Only the backbone atoms were chosen, as these are common in the wt Cas9 and mutants. For details, please refer to the Materials and Methods section. L1 (residues 765–778) and L2 (residues 907–916) linkers connect the HNH and RuvC domains, enabling an information highway between the two endonuclease Cas9 domains for concerted cleavage of the two DNA strands in an allosteric way that also involves correlated motions of the HNH–REC3–REC2–RuvC domains.^19,37,45^ Thus, conformational changes in the 718–1001 Cas9 region can be associated with the transition from the inactive to the active for cleavage conformations of the HNH and RuvC domains.^2,20,44^ By considering the whole Cas9 protein, or even a larger than the 718–1001 Cas9 region, no proper MSM models could be constructed out of the MD trajectories, with kinetically distinct macrostates that can be validated.

First, the time-structure-based independent component analysis (tICA) method is employed to decrease the dimensionality of the configurational space explored over the MD trajectories and remove any redundant information, as in ref (46). The tICA method identifies the slowest degrees of freedom, which in this case are associated with the torsional angles of the following Cas9 residues: 718, 719, 765, 768, 773, 774, 777, 779, 825, 826, 842, 864, 892, 899, 901, 907, 913, 917, 974, 976, 983, and 1001. These residues belong to the RuvC domain (31.8%), the HNH domain (36.4%), the L1 linker (22.7%), and the L2 linker (9.1%). Interestingly, the slowest degrees of freedom that can be associated with the long-term conformational changes in this region are attributed mainly to the L1 domain dynamics if we consider that this linker has a considerably smaller presence in the 718–1001 region (∼5%). This indicates that mutations in the bridge region of Cas9 and Mg^2+^ ions primarily affect the conformation of the L1 linker. Please note that the residues Gln768, His840, Arg976, and His983 are all included as important contributions to the tICA components.

**Figure 2:**
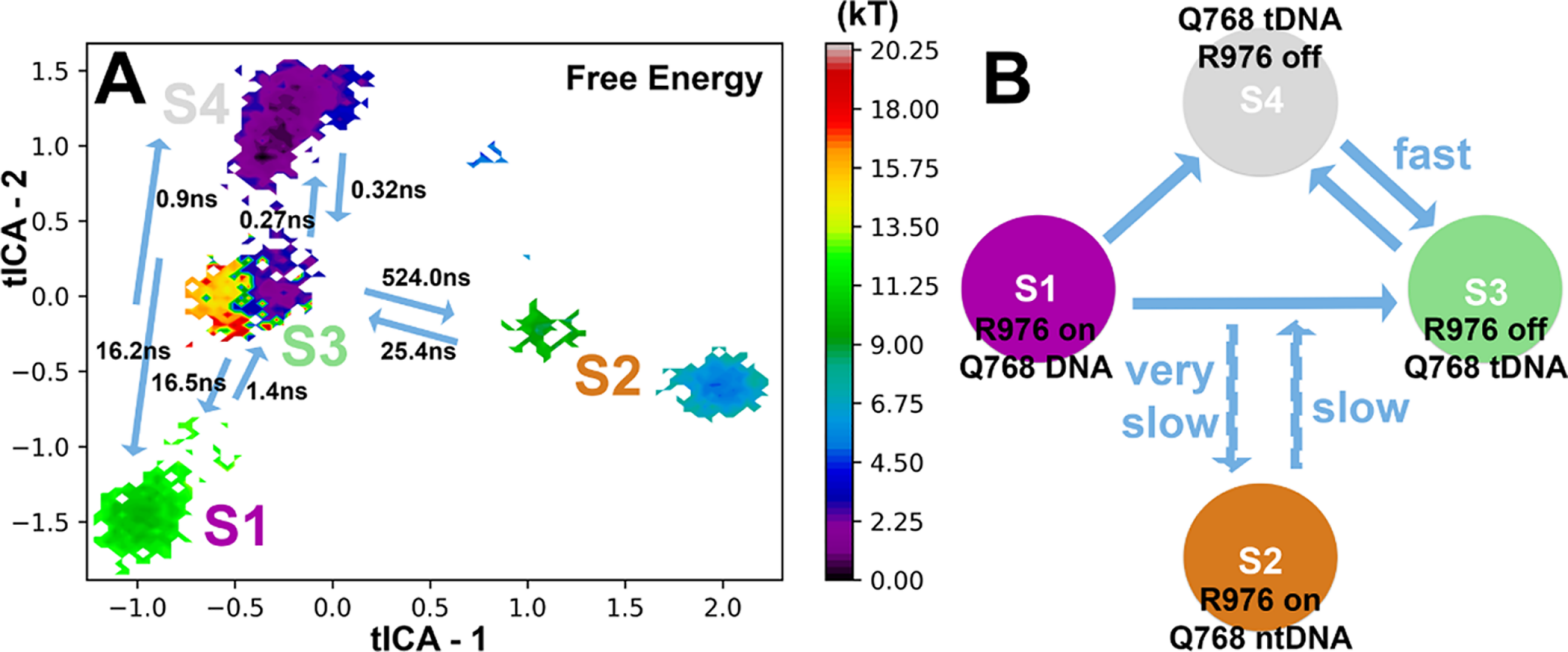
(A) Weighted free energy surface of the Cas9 718–1001 residue backbone. The position of the associated macrostates (S1–S4) is also provided. Energy values are in *kT*, with *k* being the Boltzmann constant and *T* being the temperature. Transition rates between states are provided for reference. Blue arrows indicate the direction of transition. (B) Schematic representation of the main transitions between MSM states. “R976 on/off” labels refer to the active/inactive conformations of Arg976, “Q768 DNA” label refers to the Gln768 conformation with the side chain to interact with both tDNA and ntDNA, and “Q768 tDNA” and “Q768 ntDNA” labels refer to the Gln768 conformation with the side chain to interact only with either tDNA or ntDNA, respectively.

**Figure 3:**
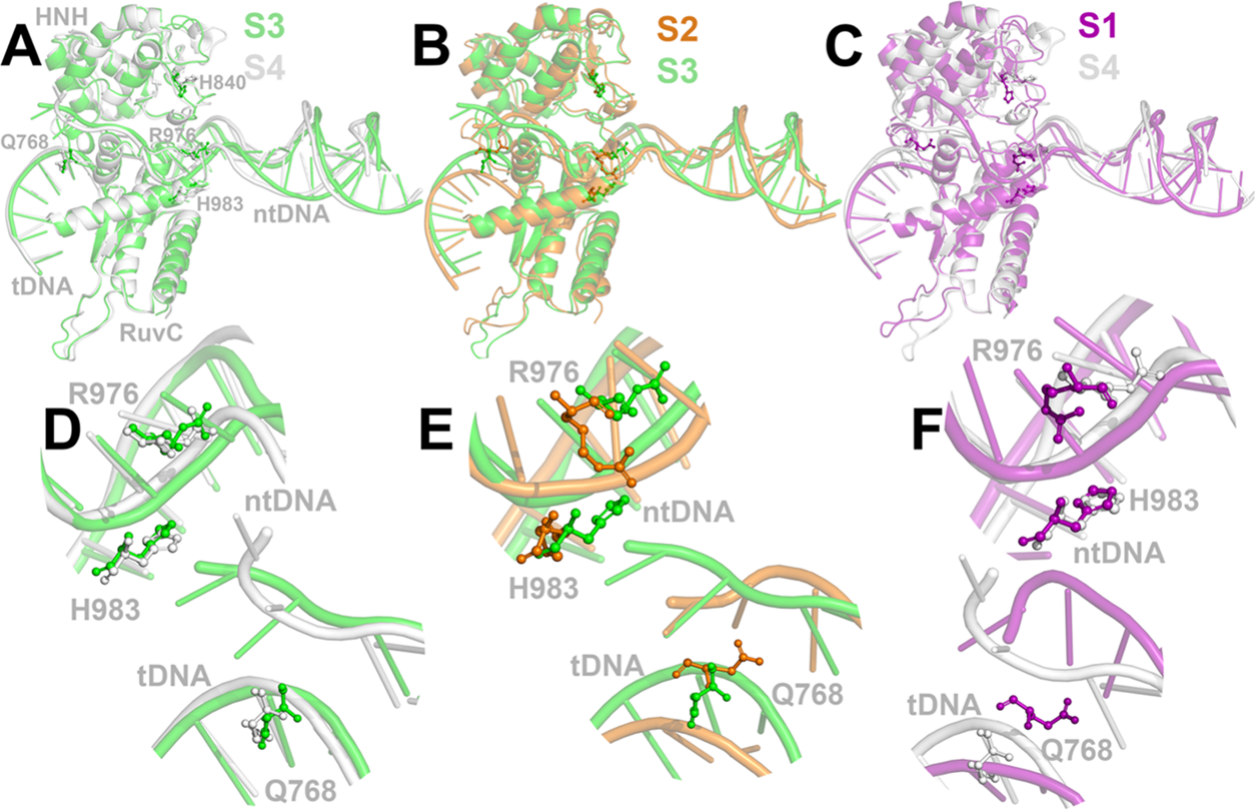
(A–C) Different conformations of the Cas9 HNH; RuvC domains; and the associated His840 (H840), His983 (H983), Gln768 (Q768), and Arg976 (R976) side chain configurations. Target (tDNA) and nontarget (ntDNA) DNA strands are also shown in cartoon representation. (D–F) Zoom into the specific regions of interest of the Cas9–sgRNA–dsDNA system.

The reweighted free energy surface (FES) of the Cas9 718–1001 region, based on the MSM analysis of all equilibrium trajectories (6.0 μs), projected on a space of torsional features (tICA components) is shown in Figure 2A, along with the position of each macrostate (S1–S4) identified. State S4 is of the lowest energy. The transition rates have been calculated between the macrostates based on the MSM model (Figure 2A). Blue arrows indicate the transition direction that is accompanied by the transition time scale (ns). Not all possible transitions are shown for clarity, and a cumulative rate time has been presented for each kind of transition. A rough schematic representation is also shown in Figure 2B. Transitions between S1–S3–S4 are feasible, while state S2 seems the least accessible (disconnected). S1 and S3 transition to S4 within short time scales (<1 ns), while S3 and S4 are in an equilibrium characterized by the shortest bidirectional transition times (0.27 and 0.32 ns). The MSM-based Cas9 macrostate conformations of the 718–1001 backbone have been associated with conformations of the whole Cas9 protein scaffold out of the classical MD trajectories (for details, please refer to the Materials and Methods section). The resulting four Cas9 conformations (macrostates, or simply states) are shown in Figure 3. The most pronounced changes in the HNH position are between states S1 and S4, as also depicted by the change of the His840 side chain position (Figure 3C). HNH exerts a conformational heterogeneity prior to its activation for cleavage.^13,44^ From an inactive conformation, the HNH domain assumes the fully active conformation close to the scissile phosphate of the target site, upon an approximately anticlockwise 140° (∼34 Å) rotation relative to the axis perpendicular to the sgRNA–DNA hybrid duplex.^13,24,45^ The HNH conformational space is considerably restricted in the presence of mismatches in the PAM-distal ends of the sgRNA–DNA hybrid duplex and locked in a “conformational checkpoint” between dsDNA binding and cleavage;^35,44^ however, partial activation of HNH is also possible.^8,12^ For the latter, His840 must come closer to the scissile phosphate. In S4, the catalytic His840 residue, along with the whole HNH domain, is sampled closer to tsDNA, compared to the S1 state (Figure 3C). Although this configuration is still at the HNH “checkpoint” regime, the MSM model has captured the transition to the partially active conformation. The respective conformations of Arg976 and Gln768 for all S1–S4 states are mapped on the rough schematic representation of Figure 2B. The Gln768 side chain is found to interact with tDNA for states S3 and S4 (Figure 3D), whereas for the S1 state, the Gln768 side chain is found to interact with both tDNA–ntDNA (Figure 3F). For state S2, the Gln768 side chain is found to interact with ntDNA (Figure 3E). The S4 state also exerts a distinct structure of the PAM-distal end of the ntDNA, compared to the other states. The Arg976 side chain interacts with His983 at states S1 and S2 (Figure 3E,F), whereas for S3 and S4, the Arg976 side chain swings away toward the HNH domain (Figure 3D). These findings for Arg976 come in line with the proposed mechanism of RuvC activation in the literature.^20^

Taking all of these results and published literature together, we can identify two main pathways on the Cas9 reaction coordinate sampled over the MD trajectories and predicted by the MSM analysis: S1 → S3 and S1 → S4 → S3. The Gln768 conformation seems to be the defining turning point. The Gln768–tDNA interactions are crucial for the identification of mismatches.^21^ If Gln768 strongly interacts with tDNA, then Arg976 switches to the “inactive” (“off”) conformation, as summarized in Figure 2B. If Gln768 interacts with either both ntDNA and tDNA or only ntDNA, then Arg976 switches to the “active” (“on”) conformation. The advantage of a Q768A–R63A dual mutant with improved specificity in Cas9^21^ could be due to the elimination of the S3–S4 states in the Cas9 reaction pathway, without a residue in the position 768 with a side chain to be able to lock on tDNA (see below on the effect of the Gln768 lock on tDNA and the stabilization of the R-loop).

**Figure 4:**
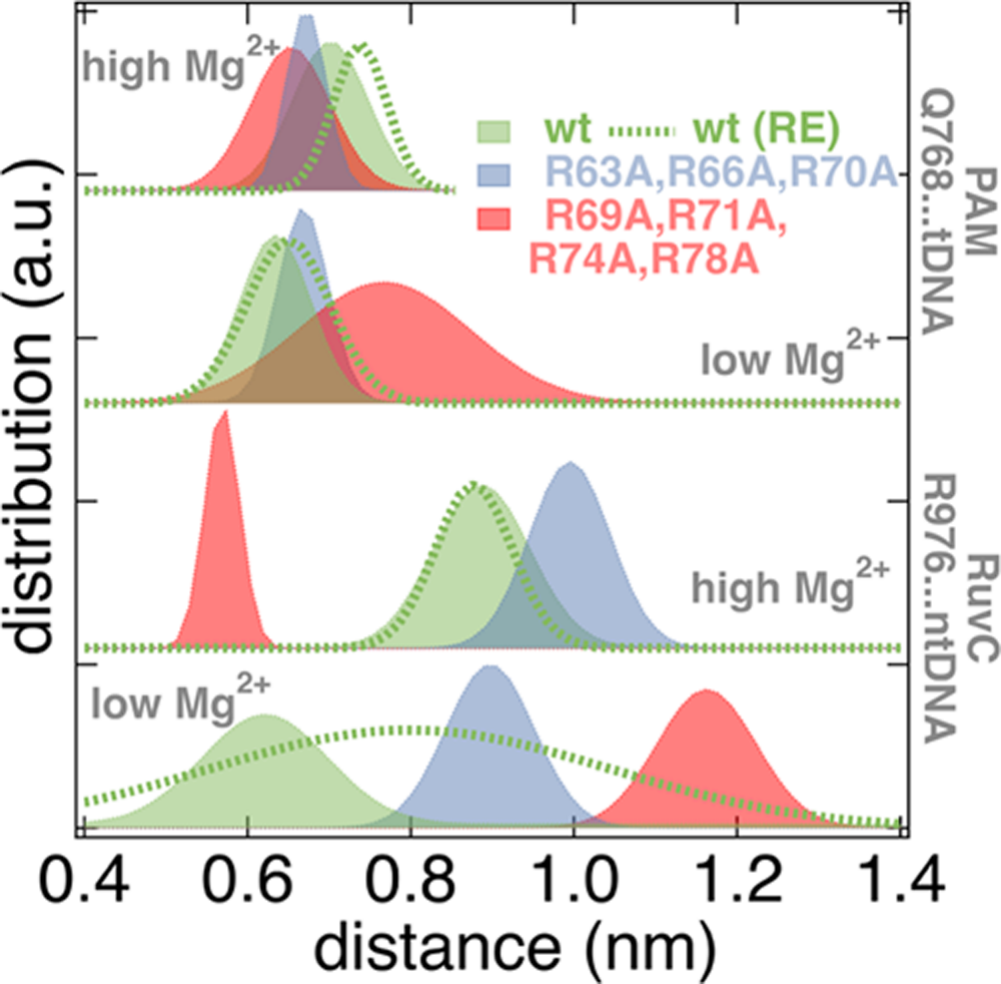
Distributions (histograms) for distances between Gln768 (Q768)–tDNA and Arg976 (R976)–ntDNA for the Cas9 variants at the low and high Mg^2+^ concentrations probed. RE (dashed green lines) refers to the Hamiltonian replica exchange results. All other distances are calculated for the classical MD trajectories. Gaussian fits have been applied for all histogram distributions.

Distributions of the distances between Gln768 and tDNA and between Arg976 and ntDNA are provided in Figure 4 for the different models probed in this study. For more distances within the Cas9–sgRNA–dsDNA system probed by classical MD trajectories, please refer to Figure S3. Please note that in the active conformation both His983 and Arg976 should approach the scissile phosphate of ntDNA, so the Arg976–ntDNA distance should be shorter compared to the inactive conformation. A considerable effect of Mg^2+^ concentration on the profiles of Figure 4 can be identified. The effect of mutations and Mg^2+^ concentration is more pronounced on the conformation of Arg976. The R63A–R66A–R70A mutation seems to be largely unaffected by the increased Mg^2+^ concentration. On the contrary, the conformations of Gln768/Arg976 in the R69A–R71A–R74A–R78A mutation are very sensitive to the Mg^2+^ concentration, exerted as shifts or histogram widths (dispersion) in the distributions of their distances to n(t)DNA. It seems that decreased specificity of Cas9 (R69A–R71A–R74A–R78A mutant)^21^ is Mg^2+^-dependent, whereas variants with increased specificity (R63A–R66A–R70A mutant)^21^ should exert less dependence on the Mg^2+^ concentration. Here, MD data and MSM models are used to show that in analogy to the “active” and “inactive” conformations of Arg976,^20^ Gln768 exerts similar behavior, with two states: one with strong interaction with tDNA and another interacting with either both ntDNA and tDNA or only ntDNA. The Arg976/Gln768 behavior seems to be dependent on mutations in the bridge residues and the Mg^2+^ concentration. Two associated videos of the motion of the Gln768 side chain are provided for reference as Supporting Information (SI).

### Enhanced Sampling and Machine Learning

**Figure 5:**
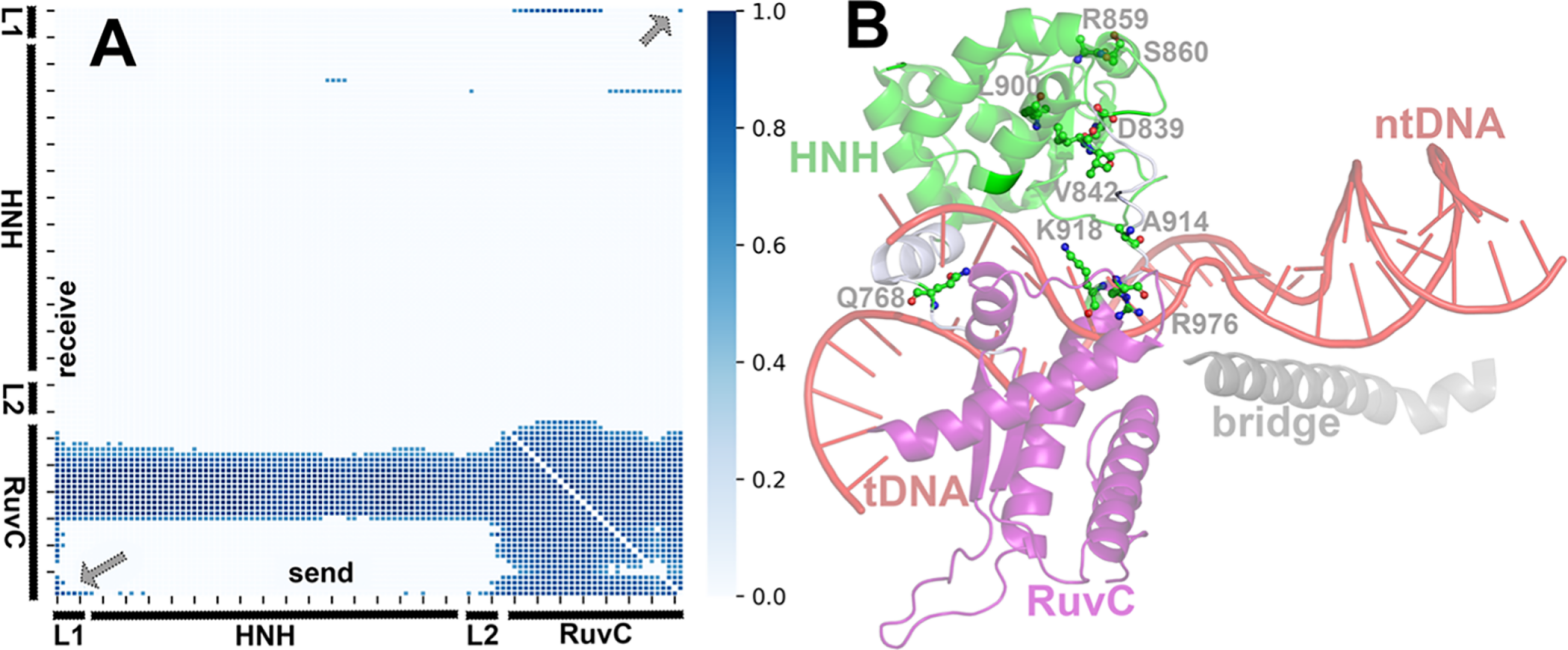
(A) Correlations for the Cas9 residues in the region 767–984. Blue gradients indicate the strength of correlations. Gray arrows designate the correlation between Gln768 and Arg976. The horizontal axis represents the domains that send information, and the vertical axis represents the domains that receive this information. (B) Position of the most important residues as defined by the shortest pathway that mediates allostery in the structure of Cas9 predicted by Neural relational inference. Only the HNH, RuvC, and bridge domains are shown and labeled with labels of the same color. Ile841 next to Val842 (V842) has been omitted for clarity.

The enhanced sampling technique of Hamiltonian Replica exchange with solute tempering (REST2) is further employed^27,28^ that enables sampling of an extensive configurational space of Cas9. dsDNA has been defined as the solute that is sampled at different effective temperatures in the 303–450 K range, so the nonbonded interactions between Cas9–sgRNA/dsDNA are used as the reaction coordinate. The method used achieves a broad sampling of the conformational space of Cas9 with transitions that depend on the nonbonded interactions (e.g., Arg976–ntDNA, Q768–n(t)DNA). A major advantage of our computational approach is that the Cas9–sgRNA–dsDNA system can effectively transition between different intermediates separated by energy barriers; thus, accelerating sampling is achieved at long time scales. The REST2 method was employed only for the wt Cas9 at low and high Mg^2+^ concentrations. Two resulting trajectories (100 ns each) were combined and analyzed with the Arg976/Gln768 profiles shown in Figure 4 (dashed green line). An elaborate analysis was performed on these trajectories by machine learning: a neural relational inference model (NRI) based on a graph neural network (GNN).^29−31^ The algorithm can predict important latent interactions between residues at long time scales by reconstructing MD trajectories of proteins. The latter approach is ideal for too short time scale simulations and predicts the time-related dynamics closely associated with the spatially long-range intraprotein communications or allostery. Within the graph theory, each residue in the protein is a node in the network. An edge between two nodes exists if the Ca atoms of the residues are within a cutoff distance of each other. A communication pathway thus is formulated within the protein scaffold. The long-range allosteric interactions within this scheme have been identified only for a small Cas9 region between residues 767 and 984 (218 residues, Ca atoms) that contains Gln768, His840, Arg976, and His983. The results are shown in Figure 5A expressed as cross correlations at the residue level.

### Implications for the Gln768–Arg976 Correlation

**Figure 6:**
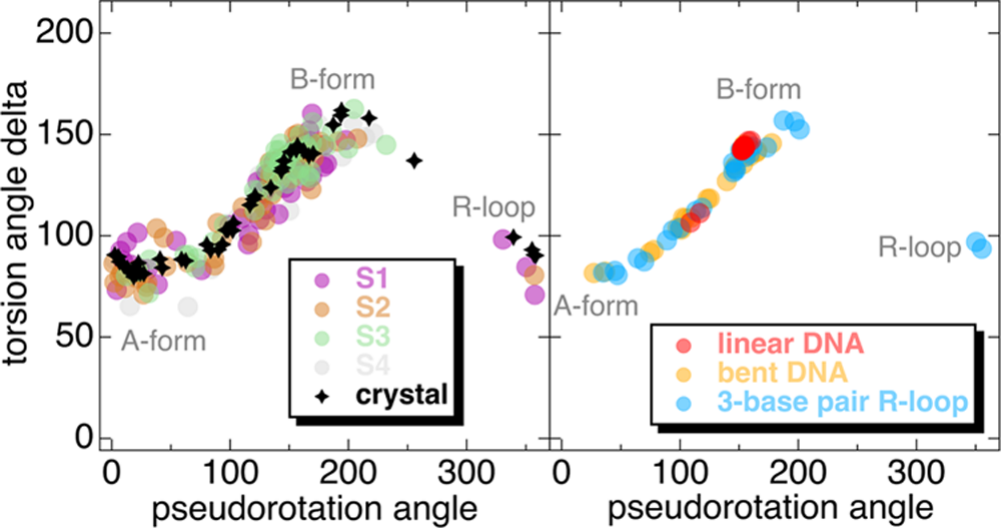
Left panel: correlation between the DNA torsion angle delta and the sugar pseudorotation angle for all MSM-defined macrostates. The black stars refer to the Cas9 crystal structure (pdb ref: 5f9r). Right panel: correlation between the DNA torsion angle delta and the sugar pseudorotation angle for the crystal structures of Cas9–sgRNA–DNA (pdb refs: 7s3h, 7s36, 7s38). Clusters of points are referred to A- or B-form and R-loop formation as shown in both panels.

The analysis revealed that Gln768 correlates with Arg976 (Figure 5A, lower left, upper right blue-shaded areas). This comes in line with the MSM macrostates predicted (Figure 3), where the side chains of Gln768 and Arg976 exert well-defined correlated conformations. Τhe shortest pathway that mediates the allosteric communication between Gln768 and Arg976 provides valuable information for the Cas9 function and is shown in Figure 5B. The most important nodes within this pathway for the information communication between Gln768 and Arg976 are predicted as the residues: Gln768, Asp839, Ile841, Val842, Arg859, Ser860, Leu900, Ala914, Lys918, and Arg976. The Asp839, ile841, Val842, Arg859, Ser860, and Leu900 residues have already been reported as important communication nodes in the activation of the HNH domain.^37^ Based on the study by Nierzwicki et al.,^23^ residues Asp839, Ile841, and Val842 belong to the A1 allosteric site of HNH (839–856) and residues Arg859 and Ser860 belong to the A2 allosteric site of HNH, with A1–A2 HNH regions being critical hotspots for the communication from REC to the catalytic domains.^23^ A seemingly large gap exists between Gln768 and the rest of the residues in the communication pathway proposed (Figure 5B). How is this gap filled? Cas9 folds around the tracrRNA scaffold, which is part of sgRNA and is guided into the conformation able to bind dsDNA and subsequently to the active conformation for cleavage upon complementarity between crRNA and dsDNA.^1^ Upon dsDNA binding, the DNA duplex denatures (unwinds), and tsDNA forms a hybrid duplex with the complementary crRNA sequence. A distorted conformation predominantly of (pseudo) A-form has been proposed for the sgRNA–DNA hybrid.^19,22,32^ The structures of the DNA strands within our proposed S1–S4 macrostates have been analyzed by the 3DNA–DSSR tool,^47^ in terms of the correlations between delta torsion angles defined as the C5’–C4’–C3’–O sugar conformation angles^48^ and the sugar pseudorotation angles.^49^ The results are shown in Figure 6 (left pane) with the correlation of these angles to be characteristic for A- or B-DNA forms^48^ and R-loop formation. The conformation of the DNA strands in the Cas9 crystal structure (pdb: 5f9r) with the Cas9–sgRNA primed for DNA cleavage^19^ is also shown for reference as black stars in the same panel. Based on the analysis of the crystal structures of DNA in the study by Cofsky et al.,^50^ a linear DNA structure should populate the B-form regime of pseudorotation angles (>100°), a bent DNA structure should populate both the A-form (<100° pseudorotation regime) and B-form regions (Figure 6, right panel), while the formation of an R-loop should exert points also on the >270° pseudorotation angle regime (Figure 6, right panel). States S1 and S2 exert DNA strand structures like the one in the Cas9 crystal structure primed for DNA cleavage (black stars). On the contrary, the S3 and S4 states lack points in the R-loop formation regime of the pseudorotation angles, indicating R-loop destabilization or distortion. The Gln768 side chain in the S3 and S4 states of Cas9 strongly interacts with the tDNA strand. So, this should be the reason behind the destabilization of the R-loop for the studied cases. In the Cas9 crystal primed for DNA cleavage^19^ and the S1 and S2 states, Gln768 interacts with either the ntDNA strand or both ntDNA and tDNA strands and stabilizes the R-loop structure. There is thus a clear impact on the DNA structure and R-loop stability by Gln768 conformation, which might be a defining point for the allosteric communication within Cas9. In the crystal structure, Gln768 interacts with the G14–A15 bases of the ntDNA. The points with R-loop associated pseudorotation angles in Figure 6 (left panel) refer to A18–C27–G19 (S1), C20 (S2), C20–T21–G28–C30 (crystal) DNA bases, which belong to the tDNA strand, except T21 and G19 that are part of the ntDNA strand.

We must consider that the actual S1–S4 structures refer to kinetically distinct MSM-predicted macrostates and do not necessarily represent time-averaged structures over the classical MD trajectories whose Gln768/Arg976 conformations exert the distance profiles in Figure 4. Classical MD trajectories could have been trapped in different minima and only transiently (if at all) sample the S1–S4 states or combinations of these. It could be also possible that different domains of the large Cas9 protein are trapped in different minima exerting profiles for the whole protein in a combination of the S1–S4 states. Thus, one can only roughly map the profiles in Figure 4 to the different MSM macrostates S1–S4, based on the position of Gln768 at low Mg^2+^ concentrations. The wt Cas9 exerts S3/S4 state behavior, the R63A–R66A–R70A mutant of increased specificity samples mainly the S1 state that transitions to S3 (Gln768 side chain interacts with tDNA and a destabilized R-loop), whereas the R69A–R71A–R74A–R78A mutant of decreased specificity samples the S2 state (Gln768 side chain interacts with ntDNA and a more stable R-loop).

## Conclusions

In conclusion, this study has revealed the role of Gln768 in the Cas9 dynamics based on the MSM analysis of MD trajectories. Gln768 switches between different conformations of its side chain (interacting with either tDNA, ntDNA, or loosely with both), which is correlated with the conformation of Arg976, a catalytic residue of the RuvC endonuclease domain of Cas9. The interaction of Gln768 with n(t)DNA strands has been determined as a key parameter in the Cas9 allosteric communications but also for the stabilization of the sgRNA–DNA hybrid R-loop structure. These findings provide an understanding of the role of Gln768, which resides at the crossroad of a communication pathway between HNH and RuvC domains. In the Q768A–R63A mutant of increased specificity, in the absence of Gln768, these communications should be disrupted with effects also on the R-loop structure. These interactions and associated allosteric communications appear as crucial for the specificity within Cas9. In detail, within the R69A–R71A–R74A–R78A mutant of decreased specificity, Gln768 fails to scan the sgRNA–tDNA hybrid duplex for mismatches and stabilizes the R-loop formation (S2 sampled state). For the R63A–R66A–R70A mutant of increased specificity, the R-loop is destabilized (in a combination of S1 and S3 sampled states). The conformational changes arising from the transitions between the different Cas9 macrostates proposed are important to gain a better understanding of the molecular determinants of Cas9 mechanism of action and provide new insight into the improvement of the CRSPR-Cas9 specificity (like in the Q768A–R63A mutant). The sampled dynamics have been compared with experimental studies. Notably, they fit well the important aspects of Cas9 function and the mutant phenotypes proposed in the literature. This work formulates the basis for further studies to characterize the effect of mutations in Cas9 and adds to the atomic-scale understanding of this powerful gene editing tool. We must note that understanding how mutations affect the Cas9 activation is per se important to decipher the Cas9 mechanism of action. The methodology setup employed herein, especially the combination of short, enhanced sampling trajectories with machine learning algorithms, can formulate the basis for future studies on the conformational space of large biomolecules.

## Materials and Methods

### System Setup

The initial Cas9 coordinates come from the most complete X-ray structure of the Cas9–sgRNA–dsDNA complex in the inactive form, without Mg^2+^ ions (pdb: 5F9R).^19^ The required coordinating Mg^2+^ ions are added by comparison (structural alignment) to the Mg^2+^-containing Cas9 structure in the literature (pdb: 4UN3).^51^ An additional Mg^2+^ ion is added, coordinating His983 that is protonated at the *N*ε position as proposed elsewhere and coordinates a water molecule that approaches the scissile phosphate and Mg-A in the active RuvC conformation.^20^ His-113, -160, -167, -840, -930, and -985 are protonated at the *N*ε site, while the rest of His residues are protonated at the *N*_δ_ site. Glu-223 and 232 are treated as protonated, while the rest of Glu residues are deprotonated. The Amber ff14sb force field^52^ has been employed for the protein, which includes the ff99bsc0 + χOL3 parameters for RNA^53^ and the OL15 parameters for DNA.^54^ For the Mg^2+^ ions, the Aqvist parameters have been implemented, as proposed elsewhere.^20,55^ The Cas9–sgRNA-dsDNA system that contains a low concentration of Mg^2+^ (3 mM) was hydrated by around 191 300 Tip3p water molecules,^56^ including all crystallographic ones. KCl at ∼150 mM concentration was added, with a ∼35 mM K^+^ surplus to neutralize the system. The Cas9 mutations (variants R63A–R66A–R70A and R69A–R71A–R74A–R78A) were prepared by the Schrodinger Maestro platform (Schrödinger Release 2022-2: Maestro, Schrödinger, LLC, New York, NY, 2021) based on the same Cas9 structure (pdb: 5F9R) as that used for the wt Cas9. Thus, three different systems were built of around 602 800 atoms each in a cubic unit cell of 18.3 nm^3^ volume. For another three systems, a much higher concentration of Mg^2+^ than physiologically relevant^57^ was introduced into the system by replacing the ∼150 mM KCl in the original three systems by ∼75 mM MgCl_2_ and a surplus of ∼17 mM Mg^2+^ to enable enhanced sampling of the Mg^2+^ ion effect on the Cas9 conformation. Cumulatively, six systems were probed (the wt Cas9 along with two mutants at low and high Mg^2+^ concentrations).

In the absence of Mg^2+^, the Cas9–sgRNA–dsDNA system is locked into the inactive conformation, as the Mg^2+^ ions are necessary to lower the energy barriers for HNH movement into the active conformation for the tsDNA cleavage.^44,58^ Mg^2+^ ions have also been implicated in the unwinding of the PAM-distal dsDNA region in an allosteric manner by increasing the energy barrier for dsDNA rewinding.^32^ In general, the Mg^2+^ ions are administered commonly in concentrations ∼10 mM along the CRISPR-Cas systems and are highly mobile within the Cas9 protein matrix and dynamically coordinated within the Cas catalytic sites. Thus, Mg^2+^ ions are known to stabilize cleavage-activated conformations, like the hybrid sgRNA–DNA intermediate at the PAM-distal site, in an allosteric but also concentration-dependent manner.^32,57^ Herein, we have probed a low Mg^2+^ concentration (3 mM) where all of the Mg^2+^ ions are placed at key sites proposed in the literature^20^ or resolved in the crystal structure.^51^ These Mg^2+^ ions exert very low mobility throughout the trajectories and simulate the physiological state of metal coordination within Cas9 (∼10 mM).^51^ On the other hand, an increased concentration of Mg^2+^ is used to “trap” Cas9 in different conformations or enhance the transition between them in relation to the mutations studied.

### Molecular Dynamics

The all-atom models, as defined previously, were used for the all-atom molecular dynamics simulations. Based on published protocols,^46,59^ all models were relaxed and equilibrated with gradual removal of constraints on the protein backbone-heavy atoms. In a series of constant-volume *n*VT and constant-pressure *n*PT ensembles, the temperature increased from 100 to 303 K,^46,59^ prior to the production runs. For the production of classical MD simulations, Newton’s equations of motion were integrated with a time step of 2.0 fs. The leap-frog integrator in GROMACS 2021 was employed.^60^ The production runs were performed in the constant-pressure *n*PT ensemble with isotropic couplings (compressibility at 4.5 × 10^–5^). van der Waals interactions were smoothly switched to zero between 1.0 and 1.2 nm with the Verlet cutoff scheme. Electrostatic interactions were truncated at 1.2 nm (short range), and long-range contributions were computed within the PME approximation.^61,62^ All hydrogen–heavy atom bond lengths were constrained by employing the LINCS algorithm.^63^ The v-rescale thermostat^64^ was employed (303 K, temperature coupling constant 0.5), and the Parrinello–Rahman barostat^65,66^ (1 atm, pressure coupling constant 2.0) was used for one trajectory of 1.0 μs per model (total of 6.0 μs). Instead of running multiple replicas of the same system (wild-type Cas9), we chose to perturb the Cas9 conformation in terms of mutations in key residues and by different Mg^2+^ concentrations. Thus, in this consensus, we probed six replicas of the Cas9 system at the classical MD level.

### Markov State Model

To analyze the 6.0 μs classical MD trajectories of the Cas9 system, only the Cas9 protein backbone was extracted, without protons, nucleic acids, water, or ions. Trajectory frames were taken every 1 ns. The frames in all of the trajectories were structurally aligned on the same reference initial structure, based on Ca-fitting with PyMOL 2.5 (Schrödinger, L., & DeLano, W.), to assure consistency in the analysis. MSMs of the Cas9 backbone were constructed from the trajectories of 6.0 μs total time using the PyEMMA package in Jupyter notebooks.^67^ Only the torsional angles of the residues 718–1001 (HNH, part of RuvC domains, and the L1/L2 linkers) were selected as the initial input features for model construction. A lag time of 50 ns and four tICA eigenvectors (dimensions) were chosen based on the VAMP2 scores^68^ to identify a set of the slowest modes among all of the initial input features.^69^ These constitute a linearly optimal combination of input features that maximizes their kinetic variance. A threshold of 0.195 was used for the contributions of each feature to the slowest degrees of freedom (tICA components). Below this threshold, the contributions (and the associated residues) were ignored. This threshold maximizes the VAMP2 score^69−71^ and is the largest threshold to include important residues like Gln768, His840, Arg976, and His983.

The conformations of the system were projected on these slowest modes as defined by the tICA method;^69^ then, the trajectory frames were clustered into 100 cluster centers (macrostates) by k-means clustering, as implemented in PyEMMA.^67^ The optimum number of macrostates (four) was proposed based on the VAMP2 score.^68^ Conformational changes of a system can be simulated as a Markov chain if the transitions between the different conformations are sampled at long enough time intervals so that each transition is Markovian. This means that a transition from one conformation to another is independent of the previous transitions. The uncertainty bounds were computed using a Bayesian scheme.^72,73^ The slowest implied time scales (three) converged quickly and were constant within a 95% confidence interval for lag times above 40 ns (Figure S1). The validation procedure is a standard approach in the MSM field. A lag time of 50 ns was selected for Bayesian model construction, and the resulting models were validated by the Chapman–Kolmogorov (CK) test (Figure S2). The CK test indicates that predictions from the built MSM (blue dotted lines) agree well with MSMs estimated with longer lag times (black lines). Thus, the model can describe well the long-time-scale behavior of our system within error (blue-shaded areas). Subsequently, the resulting MSMs were further coarse-grained into a smaller number of four metastable states or macrostates using PCCA++ as implemented in PyEMMA.^67^ Both the convergence of the implied time scales and the CK test confirm the validity and convergence of the MSM.

To associate the MSM-derived macrostates with conformations of the whole Cas9 protein, a clustering analysis was performed (Jarvis–Patrick method) on the 6.0 μs equilibrium trajectories considering the whole Cas9 protein scaffold this time. Average structures of the most populous clusters were extracted and associated with the four MSM macrostates based on the minimum root-mean-square deviation (RMSD) between their backbone atoms. State 1 (S1) belongs to the R69A–R71A–R74A–R78A mutant trajectory at high [Mg^2+^] (cluster weight at 13.7%, RMSD at 0.843 Å), State 2 (S2) belongs to the wt Cas9 trajectory at high [Mg^2+^] (cluster weight at 68.7%, RMSD at 0.707 Å), State 3 (S3) belongs to the R69A–R71A–R74A–R78A mutant trajectory at low [Mg^2+^] (cluster weight at 22.2%, RMSD at 0.980 Å), and finally State 4 (S4) belongs to the R69A–R71A–R74A–R78A mutant trajectory at low [Mg^2+^] (cluster weight at 10.4%, RMSD at 0.799 Å). Please note that structures with the lowest RMSD distances were chosen. Three structures (S1 and S3–S4) belong to the trajectory of the same mutant (R69A–R71A–R74A–R78A), although with different Mg^2+^ concentrations. This falls within our scope to sample the configurational space of Cas9 by perturbation of the Cas9 backbone by mutations or Mg^2+^. The R63A–R66A–R70A of increased specificity provided no matching structure for the S1–S4 macrostates predicted. Thus, this Cas9 mutant might be sampling a completely different configurational space with uncorrelated structures from those of the wt Cas9 or R69A–R71A–R74A–R78A mutant, although the MD data (Figure 4) indicated that this Cas9 mutant samples a combination of the S1 and S3 states.

### Hamiltonian Replica Exchange Enhanced sampling

The enhanced sampling method of Hamiltonian Replica Exchange with Solute Tempering (REST2)^27,28,74,75^ was employed to probe the Cas9–sgRNA–dsDNA interactions and induce conformational changes in the protein associated with the mechanism of action. Within the REST2 method, several replicas of the system (16) were simulated in parallel and independently, with biased nonbonded interactions. The number 16 has been chosen to provide a specific transition probability (∼20%) with the replicas to exchange at predefined intervals (10 000 steps, 2.0 fs time step). In the Hamiltonian variant employed herein, the replicas were simulated at the same temperature (303 K), but the nonbonded parameters for dsDNA were scaled and used as the replica coordinate at effective temperatures between 303 and 450 K. Thus, the solute (dsDNA) conformations were sampled and exchanged at different effective temperatures, while the temperature of the surrounding medium (Cas9, water, and ions) was kept constant; it could adjust to the altered Cas9–dsDNA interactions. This led to an efficient crossing of the energy barriers associated with the conformational changes of Cas9 or the formation of intermediates. The tempering of the nonbonded interactions between dsDNA and Cas9 makes it possible for the Cas9 protein to change conformation, as these interactions are highly dependent on the temperature and thus, the REST2 sampling scheme provides sampling of rare events and the crossing of energy barriers. Please note that the Cas9 bridge residues and the other key Cas9 residues probed in this study (His840, His983, Arg976, and Gln768) all interact with the dsDNA. One could include the Cas9 protein as a replica coordinate, but this would require a very large number of replicas and unreasonably long computational time. Only the wt Cas9–sgRNA–dsDNA system was simulated in REST2 runs, with two different Mg^2+^ concentrations for 100 ns each, at a cumulative simulation time of 2 × 16 × 100 ns = 3.2 μs.

### Machine Learning

Deep and unsupervised machine learning algorithms were employed for the trajectory analysis of the enhanced sampling simulations. A neural relational inference model (NRI) that is based on a graph neural network (GNN) was applied.^29−31^ By employing this algorithm, we gained a considerable increase in the accuracy of the predictions on short-time-scale trajectories compared to other algorithms.^29^ The Cas9 region 767–984 (218 residues, Ca atoms) was considered as extracted out of the trajectories of the whole protein. A further reduction in the number of nodes was considered with one node defined every second residue alternatively for two separate runs due to the size-memory limitations in the machine learning algorithm. Thus, the 767–984 region trajectories were coarse-grained into 109–110 nodes only. The data were divided into a training set (in intervals of 60), a validating set (intervals of 60), and a test set (intervals of 100). The number of time steps per sample was set to 50, the learning rate (LR) was at 0.0005 with a batch size of 1, and the LR was decayed by a factor of 0.5 every 200 epochs (500 epochs in total). The distance threshold for Ca–Ca interactions was set at 1.2 nm, and the threshold for plotting was set at 0.6 (Figure 5A).

### Trajectory Analysis—Important Parameters

Distances and dynamics in the analysis refer to Ca atoms of the Cas9 protein and the P atoms of dsDNA and sgRNA nucleic acids unless otherwise stated. In detail, the following conformational markers are monitored: (a) The distance between the catalytic His840 of HNH and the tsDNA cleavage site (between DA-17 and DC-18) that can distinguish between the active and inactive conformations of the HNH domain;^24^ (b) the Ser355–Ser867, Ser867–Asn1054, and Asp839–Lys866 distances for the HNH conformational transition between active and inactive conformations, revealed by FRET experiments,^76^ (c) the distance between His983 and the scissile phosphate of ntDNA (between DG-13 and DT-14) in the RuvC active site^20^ along with (d) the distance of the Arg976 side chain (terminal carbon) to the scissile phosphate (between DG-13 and DT-14);^20^ (e) the distance between Gln768 and the target DNA PAM-distal end (DA-24 and DT-25)^21^ along with (f) the Arg1333 and Arg1335 (PI domain) distance to PAM (DT-21, DG-22, and DG-23).^2^ These parameters are shown in the form of histograms in Figure S3.

## Supporting Information Available

MSM-implied time scales (Figure S1), Chapman–Kolmogorov test (Figure S2), histograms of key distances within the Cas9–sgRNA–dsDNA system (Figure S3), and description of the supporting videos for the Gln768 dynamics (PDF)
Wild-type Cas9 (Mov)
R69A–R71A–R74A–R78A mutant (Mov)

## Supplementary Material

pg2c00041_si_001.pdf

pg2c00041_si_002.mov

pg2c00041_si_003.mov
