# Supplementary material for: Deciphering the QR Code of the CRISPR-Cas9 System: Synergy between Gln768 (Q) and Arg976 (R)
Source: ACS Phys Chem Au. 2022 Sep 22;2(6):496–505. doi: 10.1021/acsphyschemau.2c00041 (PMC9955204; doi:10.1021/acsphyschemau.2c00041)
Supplement: Supplementary file 1 — pg2c00041_si_001.pdf [file pg2c00041_si_001.pdf]

Supporting Information for

Deciphering the QR code of the CRISPR-Cas9 system: Synergy between Gln768 (Q) and Arg976 (R)

Vangelis Daskalakis[1]*

[1]Department of Chemical Engineering, Cyprus University of Technology, 95 Eirinis Street, 3603 Limassol, Cyprus.

*Corresponding Author

E-mail: evangelos.daskalakis@cut.ac.cy, orcid: 0000-0001-8870-0850, Tel: +357 25002458

Contents:

Figure S1

Figure S2

Figure S3

Description of the videos of the Gln768 dynamics

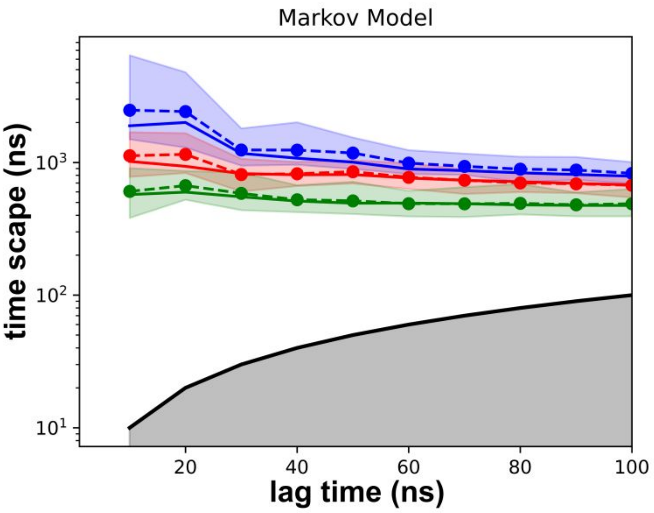

Figure S1 | The convergence behavior of the implied timescales associated with the slowest processes. The solid lines refer to the maximum likelihood result while the dashed lines show the ensemble mean computed with a Bayesian sampling procedure. The black line along with the gray shaded area indicates the timescale horizon below which the MSM cannot resolve processes.

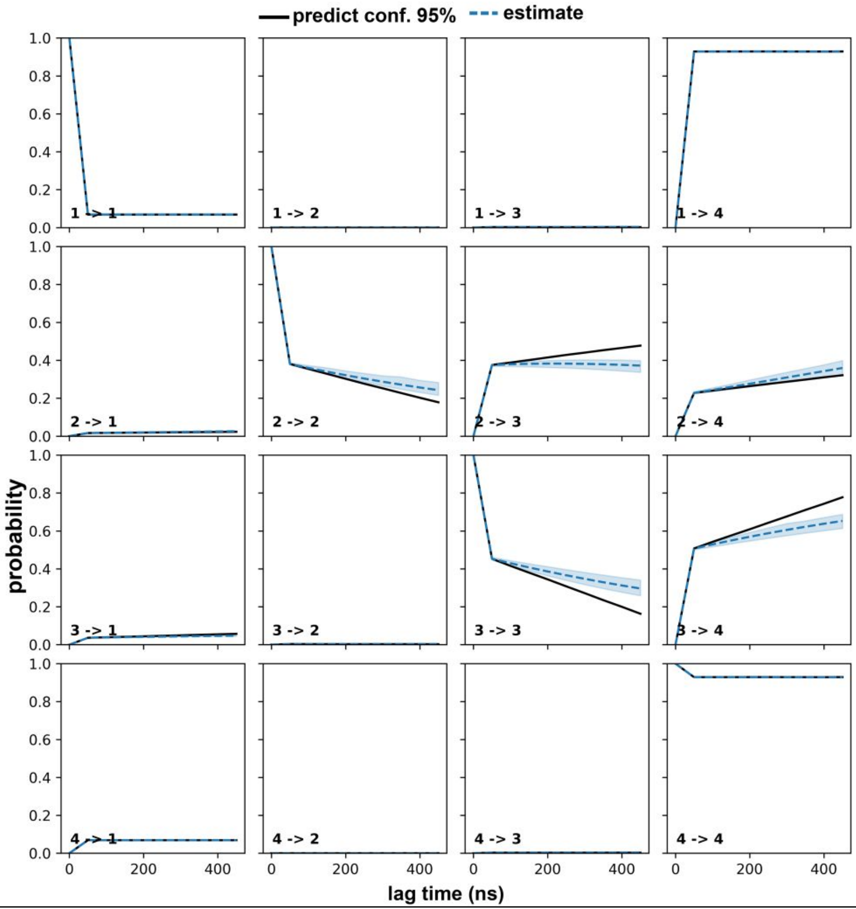

Figure S2 | The results of the Chapman-Kolmogorov (CK). The blue shaded areas indicate 95% confidence intervals computed with the Bayesian sampling procedure.

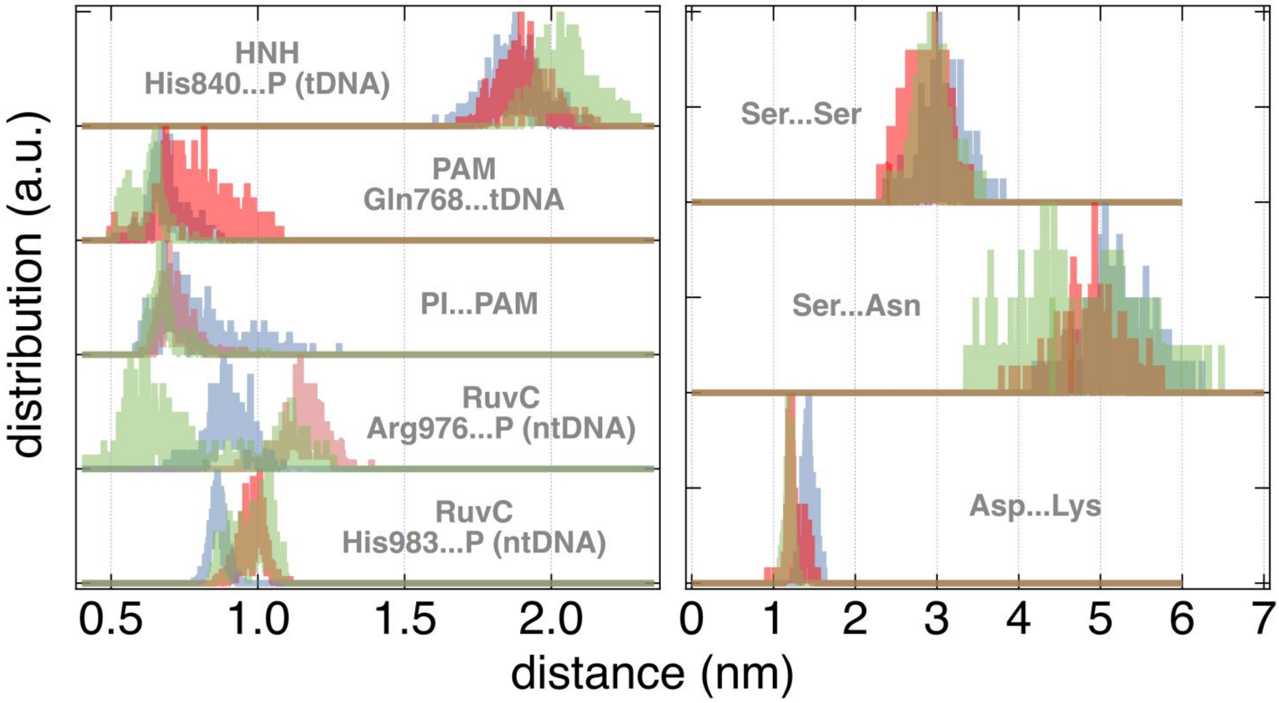

Figure S3 | The distribution of important distance within the Cas9-sgRNA-dsDNA system, as probed in the classical MD trajectories in this study for the different Cas9 variants (wt – green, R63A-R66A-R70A mutant - blue, R69A-R71A-R74A-R78A mutant - red).

Videos - Movies

Two videos are provided for the Gln768 dynamics (WT_Q768.mov for the Wild-Type and MT_Q768.mov for the R69A-R71A-R74A-R78A mutant). In these videos, Gln768 is shown as a gray tube at the end of the RuvC domain (purple cartoons) located between the non-target DNA strand (red) and the hybrid of target DNA strand (red) – RNA strand (orange). The Cas9 bridge domain is shown as green cartoon and the mutated Arg residues as gray tubes within the bridge.
